# A Single-Cell Transcriptomic Atlas of Epithelial Cell Heterogeneity During the Crown-to-Root Transition in the Mouse Molar

**DOI:** 10.3390/ijms27031162

**Published:** 2026-01-23

**Authors:** Fei Bi, Tian Chen, Jiusi Guo, Wei Qiao, Zhi Liu, Xianglong Han

**Affiliations:** 1 State Key Laboratory of Oral Diseases and National Center for Stomatology and National Clinical Research Center for Oral Diseases and National Engineering Laboratory for Oral Regenerative Medicine, West China Hospital of Stomatology, Sichuan University, Chengdu 610041, China; 2023324035079@stu.scu.edu.cn (F.B.); tchen0629@scu.edu.cn (T.C.); 2 Department of Orthodontics, West China Hospital of Stomatology, Sichuan University, Chengdu 610041, China; 3 Applied Oral Sciences & Community Dental Care, Faculty of Dentistry, The University of Hong Kong, Hong Kong SAR, China

**Keywords:** tooth root development, dental epithelium, Hertwig’s epithelial root sheath, cell heterogeneity, epithelial-mesenchymal transition, single-cell sequencing

## Abstract

The mechanisms driving the crown-to-root transition in tooth development remain incompletely understood, particularly the functional heterogeneity of dental epithelium. To address this gap and deconstruct this complexity, we aimed to analyze dental epithelial heterogeneity during this critical transition and to identify subpopulation-specific programs relevant to root development. We therefore established a single-cell transcriptomic atlas of the mouse molar at postnatal days 3.5 and 7.5, integrating 30,951 cells to profile the pan-tissue landscape and performing an in-depth analysis of 4323 dental epithelial cells. Our results reveal that the dental epithelium is composed of seven distinct subpopulations with a clear lineage hierarchy, originating from multipotent progenitors and bifurcating into self-renewing and differentiating trajectories. The identified particular functions of each subcluster include the following: structural maintaining progenitor that inhibits mineralization (Cluster 4), proliferation driver (Cluster 0), key signaling center (Cluster 1), terminally differentiated executing enamel formation (Cluster 3 and Cluster 6), and extracellular matrix-organizing hub (Cluster 5), communicating extensively via the Bmp, Tgf-β, and Wnt pathways. Our work defines dental epithelium as a dynamic and heterogeneous orchestrator of root morphogenesis, providing a foundational framework for understanding developmental biology and pioneering future regenerative strategies based on precise epithelial cell functions.

## 1. Introduction

Organogenesis, the process by which complex organs form from simpler tissues, relies on precise communication among different cell types. The developing tooth has long served as a classic model for understanding these fundamental principles [1,2], as its formation is exquisitely controlled by reciprocal interactions between epithelial and mesenchymal counterparts [3]. The mechanisms governing the development of the crown are relatively well-characterized [4], enlightening the contribution of both dental epithelial and mesenchymal cells. However, a significant gap persists in our understanding of root formation [1,5], with a disproportionate focus on mesenchymal contributions and a relative neglect of the epithelial compartment.

The dental epithelium serves as the principal orchestrator of morphogenesis throughout tooth development, instructing the underlying mesenchyme at every stage from initiation to crown patterning [6]. This guiding role is maintained during root formation [5] through the establishment of a specialized epithelial structure, Hertwig’s Epithelial Root Sheath (HERS) [7]. HERS originates from the bilayered cervical loop of the enamel organ, composed of inner and outer enamel epithelium [8], and elongates apically to delineate the future root. For decades, HERS was viewed primarily as a transient scaffold that would inevitably fragment [9,10]. This disintegration was thought to simply create a space for the dental follicle mesenchyme to populate and form the periodontal ligament and cementum [11]. However, this traditional view is being challenged by a growing body of evidence suggesting that HERS can be an active signaling center [12,13,14,15,16]. Studies have shown that epithelial-derived signals from HERS are crucial for odontoblast differentiation and dentin formation in the root [17,18,19,20]. Additionally, investigational studies implicate its direct role in periodontal ligament developing [21] and cementogenesis [22,23].

While the significance of HERS as a signaling center is now apparent, this view has largely treated it as a homogeneous entity. However, the specific epithelial subpopulations within HERS and their distinct transcriptional profiles remain poorly resolved [24]. Key questions persist: Does HERS contain dedicated progenitor cells for its apical elongation? Are there discrete, specialized subtypes responsible for signaling to the dentin-forming odontoblasts versus the cementum-forming cementoblasts? Recent investigations, primarily through single-cell RNA sequencing, have begun to illuminate this black box despite focusing on early stages of odontogenesis, suggesting that the dental epithelium harbors unexpected cellular diversity [25,26,27,28]. Resolving this heterogeneity is crucial, as the precise coordination of root formation likely depends on the temporal–spatial dynamics and intricate crosstalk of these distinct epithelial cell subsets.

To resolve the longstanding questions regarding epithelial heterogeneity and functional dynamics during the crown-to-root transition, we established a comprehensive single-cell transcriptomic atlas of the developing mouse molar epithelium. In this study, we leverage single-cell RNA sequencing of the entire mouse molar at key postnatal stages to construct a high-resolution pan-tissue atlas and deconvolute the inherent complexity of the dental epithelium. We systematically define the cellular heterogeneity, lineage dynamics, and intercellular communication networks that underpin early root formation. Furthermore, we identify and characterize a unique, functionally pivotal epithelial subpopulation with a hybrid epithelial–mesenchymal signature, revealing its central role as an extracellular matrix-organizing hub and a key signaling integrator. Our work provides an integrative framework for understanding how coordinated transcriptional programs and cellular crosstalk drive the morphogenetic transition from crown to root, offering fundamental insights into the mechanisms of organogenesis and hard tissue formation.

## 2. Results

### 2.1. Pan-Tissue Single-Cell Atlas of the Developing Mouse Molar at P3.5 and P7.5

To establish a global reference of the cellular diversity during early root formation, we performed an integrated analysis of publicly available single-cell RNA sequencing data from entire mouse molar tissues at postnatal days 3.5 (P3.5) and 7.5 (P7.5) (NCBI GEO: GSM5700360, GSM5700361) [29].

**Figure 1:**
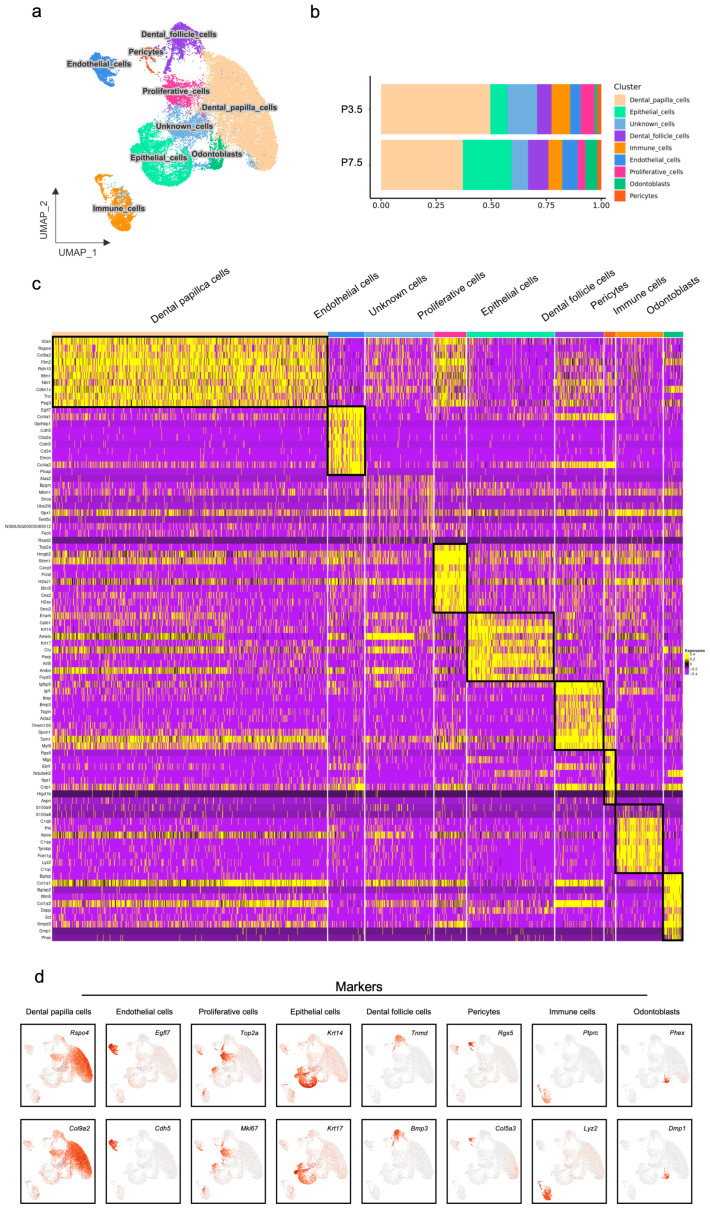
Single-cell transcriptome sequencing and integration analysis of mouse molars at P3.5 and P7.5: (**a**) UMAP plot visualizing the clusters of mouse molars; (**b**) relative proportions indicating different cell clusters in mouse molars; (**c**) heatmap of gene expression for each cell type of mouse molars; (**d**) expression patterns of the marker genes for the main cell types on the UMAP plot.

The analysis encompassed a total of 30,951 high-quality cells following rigorous quality control and data integration. Unsupervised clustering revealed nine distinct cell populations (Figure 1a). Proportional analysis from P3.5 to P7.5 (Figure 1b) displayed a marked decrease in dental papilla, immune, and proliferative cells, contrasted by an increase in dental epithelial, dental follicle, endothelial cells, and odontoblasts, while pericytes’ level remained stable. A shift pattern reflected the tissue’s transition from a proliferative to a differentiating state during early root morphogenesis. A heatmap visualizing the expression of the top ten differentially expressed genes (DEGs) for every lineage (Figure 1c) displayed the distinct transcriptional profiles of the cell types. Markers for each population were shown as follows (Figure 1d): dental papilla cells (*Rspo4*, *Col9a2*), endothelial cells (*Egfl7*, *Cdh5*), proliferative cells (*Top2a*, *Mki67*), epithelial cells (*Krt14*, *Krt17*), dental follicle cells (*Tnmd*, *Bmp3*), pericytes (*Rgs5*, *Col5a3*), immune cells (*Ptprc*, *Lyz2*), and odontoblasts (*Bglap*, *Dmp1*).

**Figure 2:**
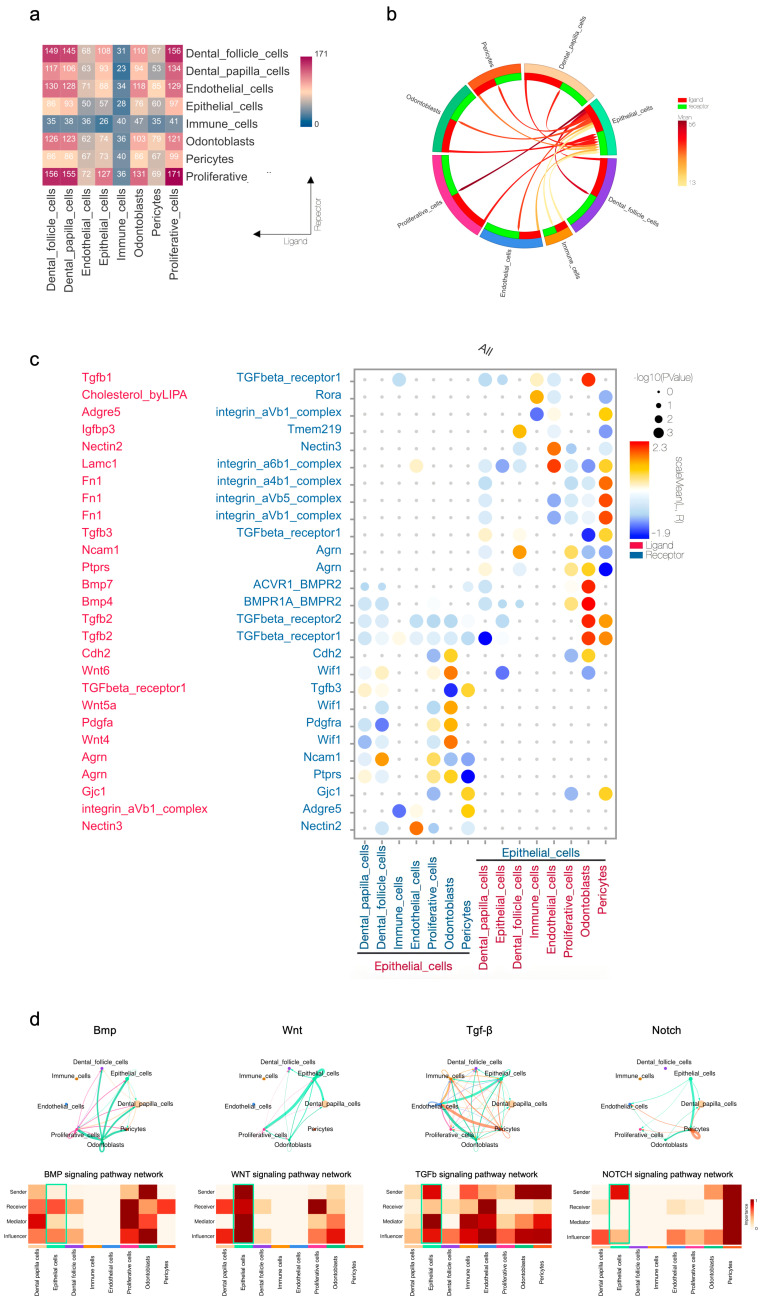
Intercellular communication focusing on the epithelial cells with the other counterparts of the mouse molar at P3.5 and P7.5: (**a**) heatmap quantifying the number of significant ligand–receptor pairs between each cluster; (**b**) circos plot with chordal graph visualizing the overall strength of intercellular communication; (**c**) bubble plot depicting the strength, significance, and specificity of ligand–receptor interactions (*p* value ≤ 0.01, Rank ≥ 0.375, Mean ≥ 0.233); (**d**) circos plot and heatmap revealing the specific role of epithelial cells in signaling pathway network analysis.

To decipher the potential interactions and signaling networks among the newly identified epithelial subpopulations and uncover the fundamental biological significance encoded within these cellular dialogues, we performed a comprehensive analysis of intercellular communication using CellPhoneDB4 and CellChat2. A heatmap depicting the number of significant ligand–receptor pairs is presented in Figure 2a. A circos plot combined with chordal graphs was employed to illustrate the overall strength of intercellular communication involving the epithelial cluster (Figure 2b), highlighting its prominent ligand–receptor interactions with other compartments by color saturation. Both of the above charts revealed that the proliferative and dental follicle cell clusters possessed the strongest interaction with the epithelial cluster. Interestingly, further investigation with detailed strength, significance, and specificity of ligand–receptor interactions, as shown by the bubble plot (Figure 2c), showed that the odontoblasts and pericytes displayed increasing intensity in interacting with the epithelial cluster, especially via Tgf-β-, Bmp-, and Wnt-related ligand–receptor pairs. The epithelial cluster could serve as the sender in Tgf-β, Wnt, and Notch signaling but the receiver in the Bmp counterpart (Figure 2d). It exhibited broad, strong connectivity with proliferative and dental follicle clusters, while engaging in specific, high-intensity crosstalk with odontoblasts and pericytes. Collectively, these data establish the dental epithelium as a central signaling hub that orchestrates complex interactions with other clusters, indicating its pivotal function in coordinating root morphogenesis.

### 2.2. Cellular Heterogeneity and Lineage Dynamics Within the Dental Epithelium

**Figure 3:**
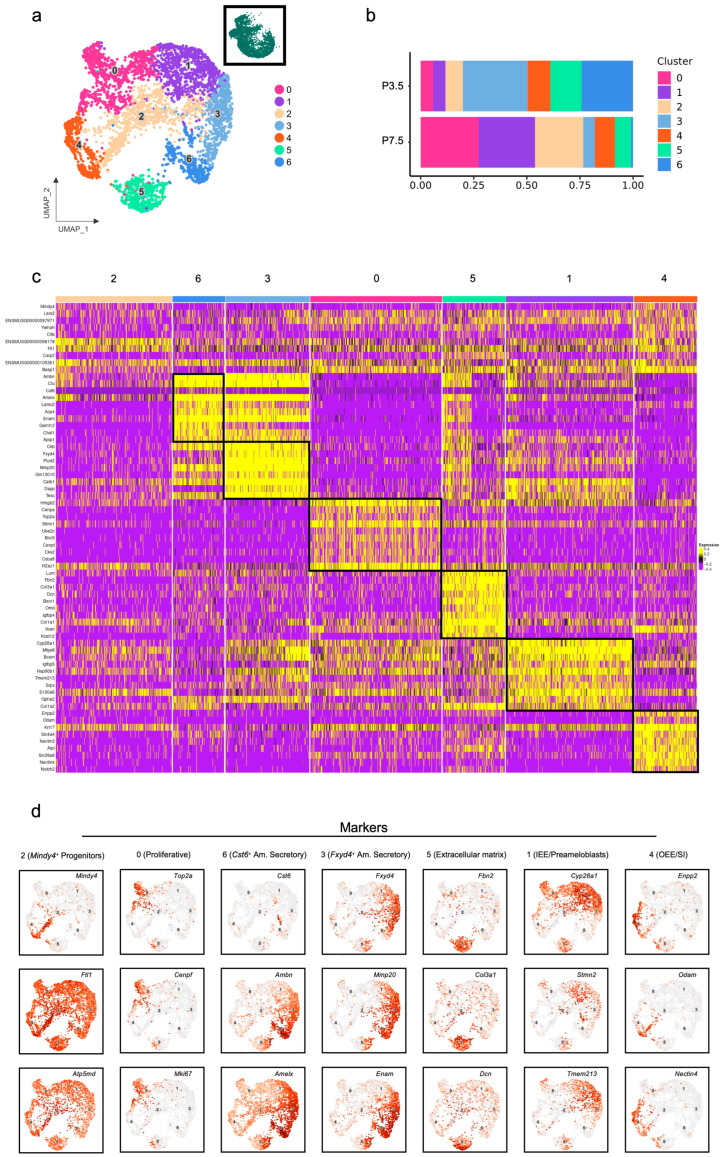
Single-cell transcriptome sequencing and integration analysis of dental epithelium in mouse molars at P3.5 and P7.5: (**a**) UMAP plot visualizing the reclustering from dental epithelium in mouse molars; (**b**) relative proportions indicating different cell clusters of dental epithelium in mouse molars; (**c**) heatmap of gene expression for each cell type of dental epithelium in mouse molars; (**d**) expression patterns of the marker genes for the cell types on the UMAP plot.

The above pan-tissue analysis provided a robust foundation for specifically exploring the heterogeneity and function of the dental epithelium during the critical crown-to-root transition. Then, the epithelial cluster containing 4323 cells was extracted for subsequent in-depth investigation. Unsupervised clustering displayed seven subpopulations (Figure 3a). Proportional analysis of epithelial subclusters from P3.5 to P7.5 (Figure 3b) revealed three distinct dynamic patterns: (1) Cluster 0, 1, and 2 exhibited a marked increase in abundance; (2) Cluster 3 and 6 underwent a pronounced decline; (3) Cluster 4 and 5 remained relatively stable. A heatmap of the top ten DEGs for each epithelial cluster (Figure 3c) provided more information about their discrete transcriptional identities. The markers for each population were as follows (Figure 3d): Cluster 0 Proliferative (*Top2a*, *Cenpf*, *Mki67*); Cluster 1 IEE/Preameloblast (*Cyp26a1*, *Stmn2*, *Tmem213*); Cluster 2 *Mindy4*^+^ Progenitor (*Mindy4*, *Ftl1*, *Atp5md*); Cluster 3 *Fxyd4^+^* Am. Secretory (*Fxyd4*, *Mmp20*, *Enam*); Cluster 4 OEE/SI (*Enpp2*, *Odam*, *Nectin4*); Cluster 5 Extracellular matrix (*Fbn2*, *Col3a1*, *Dcn*); and Cluster 6 *Cst6^+^* Am. Secretory (*Cst6*, *Ambn*, *Amelx*). Overall, during root development, the secretory ameloblast populations declined, concomitant with an expansion of the inner enamel epithelium (IEE)/preameloblasts and part of the outer enamel epithelium (OEE) compartments. This shift suggests a progressive growth of HERS, which is primarily composed of these two cell types. Furthermore, we identified a unique epithelial subpopulation characterized by the high expression of extracellular matrix (ECM)-related genes, warranting further investigation into its specific role.

**Figure 4:**
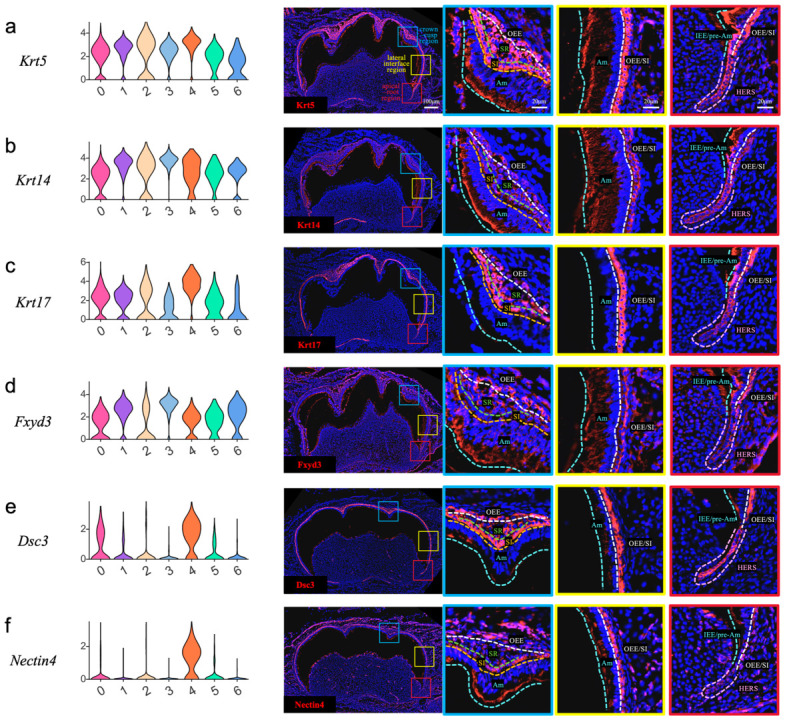

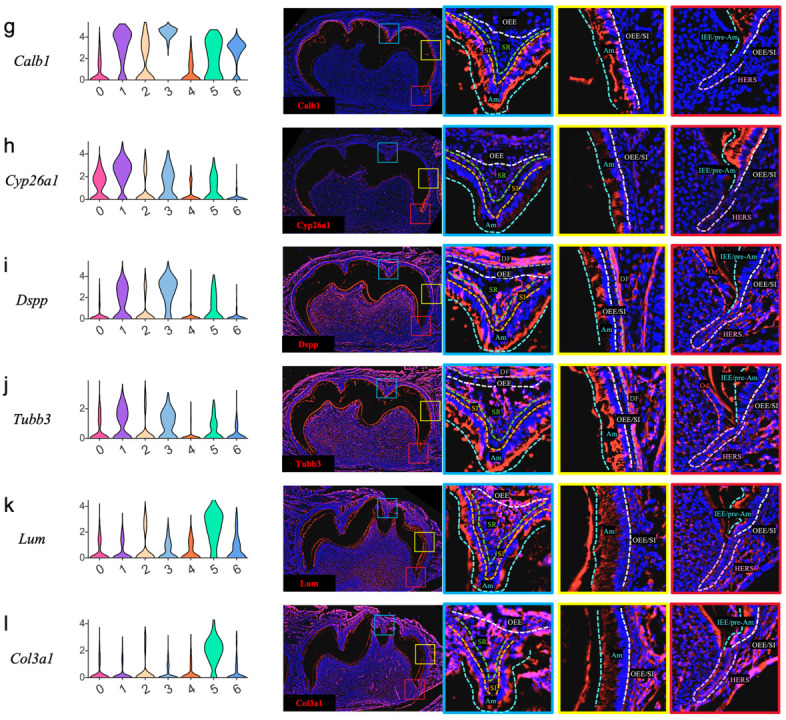
Verification of single-cell sequencing results by immunofluorescence of the mouse molar at P7.5. A violin chart displaying the expression level of marker genes and immunofluorescence staining of corresponding proteins in the mandibular first molar. The blue squares focus on the crown-cusp region containing the OEE, SR, SI, and Am. The yellow squares focus on the interface between the crown and apical side, containing the Am and the OEE/SI. The red squares focus on the cervical site and apical area containing the IEE/pre-Am, OEE/SI, and HERS. OEE—outer enamel epithelium, SR—stellate reticulum, SI—stratum intermedium, IEE—inner enamel epithelium, Am—ameloblast (Am), HERS—Hertwig’s epithelial root sheath, DF—dental follicle, Od—odontoblast. (**a**) Krt5 was broadly expressed throughout all epithelial compartments at a high level. (**b**) Krt14 exhibited broad but comparatively low expression across all epithelial compartments relative to Krt5. (**c**) Krt17 was highly expressed in the OEE, SR, and SI, but was absent in Am. Notably, within the apical region, strong KRT17 expression was also observed in the IEE/pre-Am, OEE/SI, and HERS. (**d**) Fxyd3 exhibited an expression pattern similar to Krt14, but with relatively stronger immunoreactivity specifically localized to the Am, IEE/pre-Am, and OEE. (**e**) Dsc3 was highly expressed in the OEE, SR, SI, and HERS. In contrast, only a very slight expression was detected in the Am and IEE/pre-Am layer. (**f**) Nectin4 expression was strongest in the OEE and SI, while other epithelial compartments showed only minimal detection. (**g**) Calb1 expression was predominantly restricted to the Am and IEE/pre-Am. (**h**) Cyp26a1 was expressed in a cusp-to-apical gradient within the Am and IEE/pre-Am compartments, with the strongest signal apically. Weak expression was also present in the HERS. It was not observed in other epithelial regions. (**i**) Dspp immunoreactivity was strong in the dentin matrix, Od, Am, DF, and a subset of SR cells. The signal was much weaker in the IEE/pre-Am and HERS, and was hardly detectable in the OEE and SI. (**j**) Tubb3 exhibited an expression pattern identical to Dspp, with a strong signal in the dentin, Od, Am, DF, and a subset of SR cells; weaker expression in the IEE/pre-Am and HERS; and negligible detection in the OEE and SI. (**k**) Lum was highly expressed in the Am, crown-sided OEE, IEE/pre-Am, and a subset of SR cells. In contrast, only vague immunoreactivity was detected in the apical-sided OEE/SI complex and HERS. (**l**) Col3a1 expression largely mirrored the pattern of Lum, with a strong signal in the Am, crown OEE, IEE/pre-Am, and subsets of SR cells, and faint expression in the apical OEE/SI. A key distinction was the relatively higher expression level detected in the HERS.

To validate the protein expression and spatial distribution of key epithelial subpopulations identified by single-cell RNA sequencing, we performed immunofluorescence analysis on mouse first mandibular molars at P7.5. It revealed strong, pan-epithelial expression of Krt5, confirming its role as a fundamental structural marker of the dental epithelium at this stage (Figure 4a). In contrast to the robust expression of Krt5, Krt14 was expressed at a lower level throughout the dental epithelium (Figure 4b). Expression of Krt17 marked specific epithelial compartments, showing high levels in the OEE, SR, and SI, with no detection in the Am. This pattern shifted apically, where Krt17 was additionally prominent in the IEE/pre-Am layer, OEE/SI, and HERS, suggesting a role in the structuring of the growing root (Figure 4c). Similarly to Krt14, Fxyd3 was broadly expressed throughout the epithelium. However, its signal intensity was higher in the Am, IEE/pre-Am, and OEE layer (Figure 4d). The expression domain of Dsc3 largely overlapped with that of Krt17, showing strong immunoreactivity in the OEE, SR, SI, and HERS, with markedly faint signals in the Am and IEE/pre-Am lineages (Figure 4e). Nectin4 expression was predominantly localized to the OEE and SI, with negligible detection in other epithelial compartments (Figure 4f). Calb1 was detected almost exclusively within the Am and IEE/pre-Am lineages (Figure 4g). Cyp26a1 expression displayed a distinct spatial gradient, detected in the Am and IEE/pre-Am on the apical side but largely absent from their counterparts on the crown-cusp side, with signal intensity increasing toward the apex and weak expression in HERS. It was barely detectable in all other epithelial compartments (Figure 4h). Dspp exhibited a broad yet compartmentalized expression profile. The strongest expression was localized to odontoblasts with their secreted dentin matrix, ameloblasts, and dental follicle cells, with occasional positivity in the SR. Moderate expression was seen in the IEE/pre-Am and HERS, while the OEE and SI were largely negative (Figure 4i). Similarly to Dspp, Tubb3 was strongly expressed in dentin, odontoblasts, ameloblasts, dental follicle cells, and certain cells within SR. It showed slight expression in the IEE/pre-Am and HERS, and was hardly seen in the OEE and SI (Figure 4j). Lum expression exhibited a clear regional bias. A strong signal was localized to the Am, the OEE in the coronal region, and sporadic SR cells, while expression diminished apically, appearing only faint and diffuse in the apical OEE/SI and HERS (Figure 4k). Similarly to Lum, Col3a1 was highly expressed in ameloblasts, the crown-sided OEE, and some SR cells. However, Col3a1 exhibited stronger immunoreactivity in the HERS compared to the faint signal observed for Lum (Figure 4l).

Based on the comprehensive immunofluorescence analysis, the spatial distributions of the twelve validated markers delineate distinct epithelial and mesenchymal compartments within the developing molar at P7.5. Broad epithelial expression was observed for Krt5 and Krt14, while Krt17, Fxyd3, Dsc3, and Nectin4 exhibited specific localizations to various combinations of the OEE, SR, SI, and HERS. Graded or lineage-restricted patterns were defined by Calb1 in the Am and the IEE/pre-Am and by the apical-to-cusp gradient of Cyp26a1. A unique, shared expression profile across dentin, odontoblasts, ameloblasts, and dental follicle cells was confirmed for Dspp and Tubb3. Finally, Lum and Col3a1 showed strong expression in the Am and IEE/pre-Am, with a relatively slight expression in the HERS, further defining regional matrix signatures.

**Figure 5:**
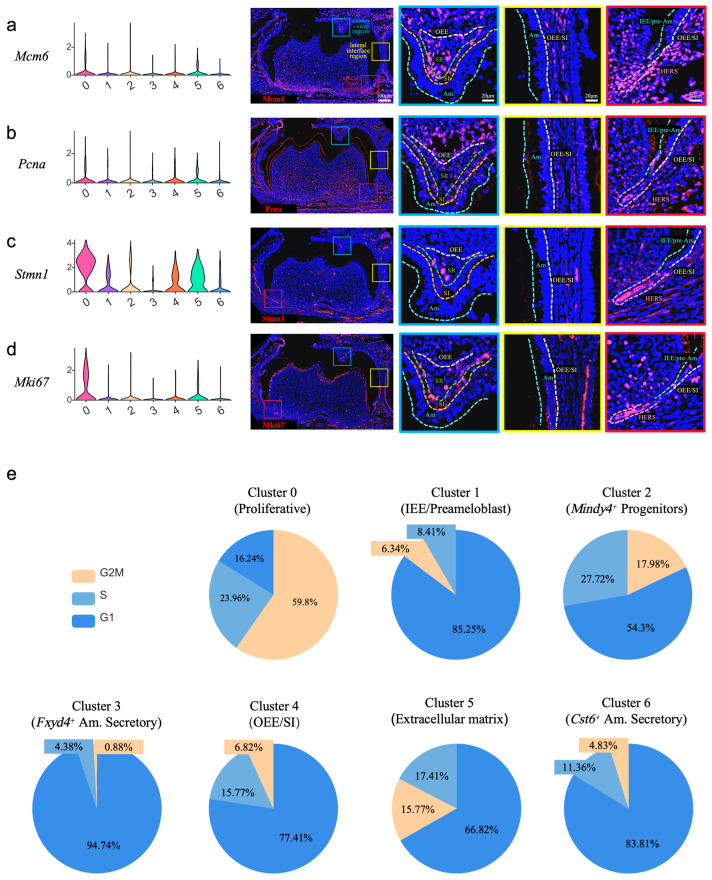
The evaluation of the proliferative potentials across the distinct epithelial subclusters. A violin chart displaying the expression level of marker genes and immunofluorescence staining of corresponding proteins in the mandibular first molar at P7.5. The blue squares focus on the crown-cusp region containing the OEE, SR, SI, and Am. The yellow squares focus on the interface between the crown and apical side containing the Am and OEE/SI. The red squares focus on the cervical site and apical area containing the IEE/pre-Am, OEE/SI, and HERS. OEE—outer enamel epithelium, SR—stellate reticulum, SI—stratum intermedium, IEE—inner enamel epithelium, Am—ameloblast, HERS—Hertwig’s epithelial root sheath. (**a**) Mcm6 exhibited scattered expression in the coronal SI, SR, and OEE, as well as in the lateral OEE/SI regions, but was absent in the Am. A strong signal was detected throughout the HERS. (**b**) Pcna showed weak expression in the coronal SI, SR, and OEE, with even lower levels in the Am. An intense signal was localized to the apical region of the HERS. (**c**) Stmn1 was highly expressed along the entire HERS and in very few scattered SR cells, but was not detected in the other epithelial compartments. (**d**) Mki67 displayed scattered expression in the coronal SI and SR, and a strong signal in the middle region of the HERS. (**e**) Pie chart showing the proportion of G1/G2M/S phases of the dental epithelial subsets of mouse molars at P3.5 and P7.5.

To complement our transcriptomic inferences of proliferative capacity and to localize these states within the native tissue architecture, we performed immunofluorescence analysis of key proliferation markers on mouse mandibular molars at P7.5. The spatial expression patterns indicated compartmentalized proliferative activity within the dental epithelium, with the HERS as the predominant proliferative hub. Mcm6, a licensing factor for DNA replication, was strongly detected throughout the HERS. Scattered expression was also observed in the coronal SI, SR, and OEE, as well as in the lateral OEE/SI regions, whereas the Am were negative (Figure 5a). Pcna, a marker of ongoing replication, showed a strong signal confined to the apical region of the HERS, with only faint expression in the coronal SI, SR, and OEE, and even weaker detection in the Am (Figure 5b). Stmn1 (Stathmin 1), which is involved in microtubule dynamics during mitosis, exhibited a highly restricted pattern, being undetectable in most epithelial compartments but strongly expressed along the entire length of the HERS and in a few scattered SR cells (Figure 5c). Finally, the canonical mitotic marker Mki67 (Ki-67) was strongly expressed in the middle region of the HERS and showed scattered positivity in the coronal SI and SR (Figure 5d). Collectively, these in situ protein-level data support and spatially contextualize the proliferative landscape inferred from single-cell transcriptomics. They identify the HERS as a major proliferative hub during root formation, with a coordinated expression of multiple replication and mitotic markers. The scattered proliferative activity within the coronal epithelial compartments (SI, SR, OEE) is consistent with the presence of progenitor pools, whereas the absence of these markers in the ameloblasts corroborates their post-mitotic, terminally differentiated state.

To quantify proliferative states at the transcriptomic level and relate them to subcluster identities, we analyzed the distribution of epithelial cells across cell cycle phases (G1, S, and G2/M) (Figure 5e). The resulting cell cycle profiles showed a clear gradient of cycling activity across subclusters and were consistent with our marker-based annotations. Cluster 0 (Proliferative Progenitors) displayed the highest cycling fractions, with 59.8% of cells in the G2/M and 24.0% in the S phase, indicating that it represents the main cycling population. Clusters 2 (Mindy4^+^ Progenitor; 18.0% G2/M, 27.7% S) and 5 (ECM-associated; 15.8% G2/M, 17.4% S) showed intermediate cycling activity. Notably, Cluster 2 had the highest S-phase fraction among all clusters, consistent with active DNA replication and a progenitor/amplifying state. Clusters 1 (IEE/Preameloblast; 6.3% G2/M, 8.4% S) and 4 (OEE/SI; 6.8% G2/M, 15.8% S) exhibited lower but detectable cycling fractions. The relatively higher S-phase component in Cluster 4 is consistent with retained replicative potential, whereas the lower cycling fractions in Cluster 1 are consistent with a more fate-committed precursor state. In contrast, the secretory ameloblast clusters showed low cycling fractions: Cluster 6 (*Cst6*^+^ Am. Secretory) had 4.8% of cells in the G2/M and 11.4% in the S phase, and Cluster 3 (*Fxyd4*^+^ Am. Secretory) was largely non-cycling (0.9% G2/M, 4.4% S).

Overall, these cell cycle distributions delineate a spectrum ranging from highly cycling cells (Cluster 0), to moderately cycling progenitor-like populations (Clusters 2 and 5), to low-cycling precursor/stem-like compartments (Clusters 1 and 4), and finally to largely non-cycling secretory ameloblast populations (Clusters 3 and 6).

**Figure 6:**
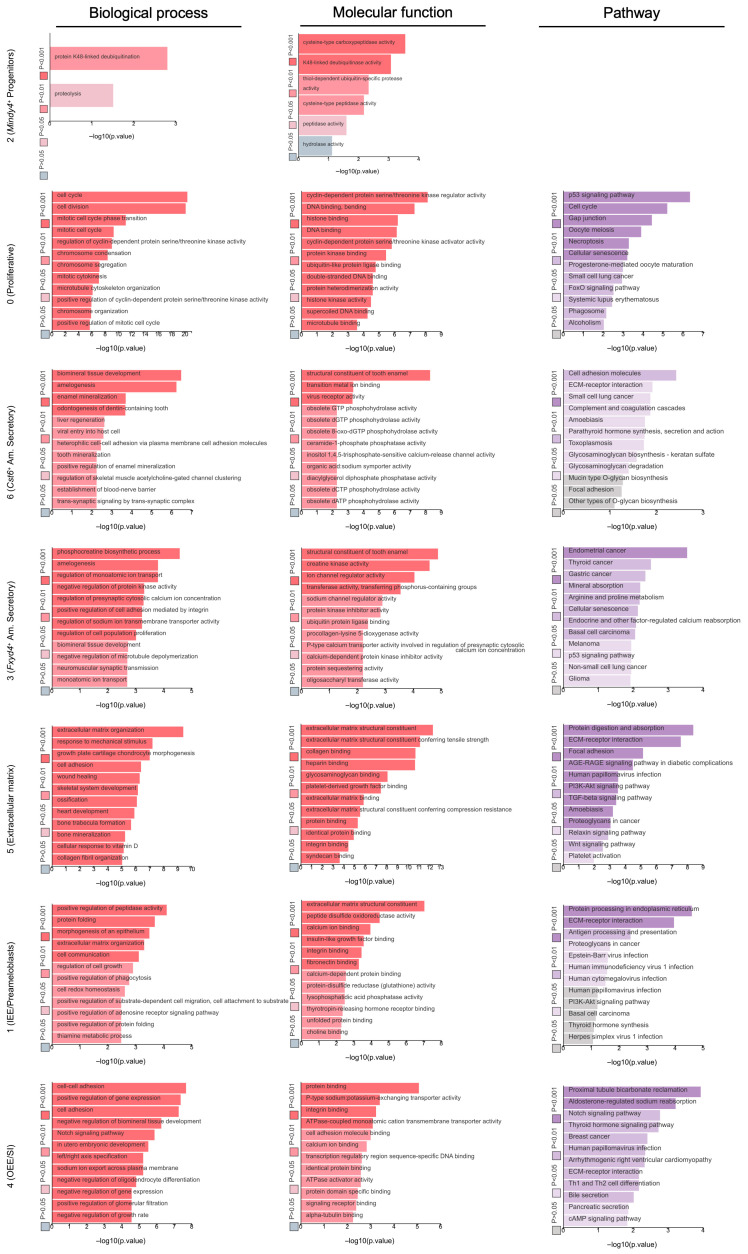
GO and KEGG pathway analyses of the dental epithelial subsets of mouse molars at P3.5 and P7.5. log2FC ≥ 1; adj *p* value ≤ 0.01.

To elucidate the distinct biological functions and signaling pathways associated with the identified epithelial subpopulations, we performed Gene Ontology (GO) and Kyoto Encyclopedia of Genes and Genomes (KEGG) pathway enrichment analyses on the DEGs of each cluster (Figure 6). The results revealed compelling functional specialization among the clusters. The transcriptional profiles of Clusters 3 and 6 were defined by terms such as amelogenesis and biomineral tissue development, positioning them as functional ameloblasts. Cluster 0 showed significant enrichment in cell cycle and cell division, identifying it as a highly proliferative progenitor population. Cluster 5 was markedly enriched for terms related to extracellular matrix organization, cell adhesion, ossification, bone trabecula formation, and bone mineralization, suggesting a role in structuring the proper microenvironment for mineralized hard tissue formation. Cluster 1 showed strong associations with terms relating to morphogenesis of an epithelium, extracellular matrix organization, and cell communication, suggesting an active key role in guiding and mediating interactions with the surrounding tissues during root formation. Cluster 4 was significantly enriched for terms related to cell–cell adhesion, cell adhesion, and the negative regulation of biomineral tissue development. This would indicate specialized roles for Cluster 4, being responsible for maintaining the structural integrity of the HERS, as well as actively inhibiting mineralization to ensure the proper interface formation between the unmineralized periodontal ligament and cementum deposited on the root surface. Furthermore, KEGG pathway analysis underscored these functional distinctions, highlighting key signaling pathways. Notably, the p53 signaling pathway and cell cycle were enriched in Cluster 0, the Notch signaling pathway was enriched in Cluster 4, while the ECM–receptor interaction, Tgf-β signaling pathway, and Wnt signaling pathway were prominent in Cluster 5.

**Figure 7:**
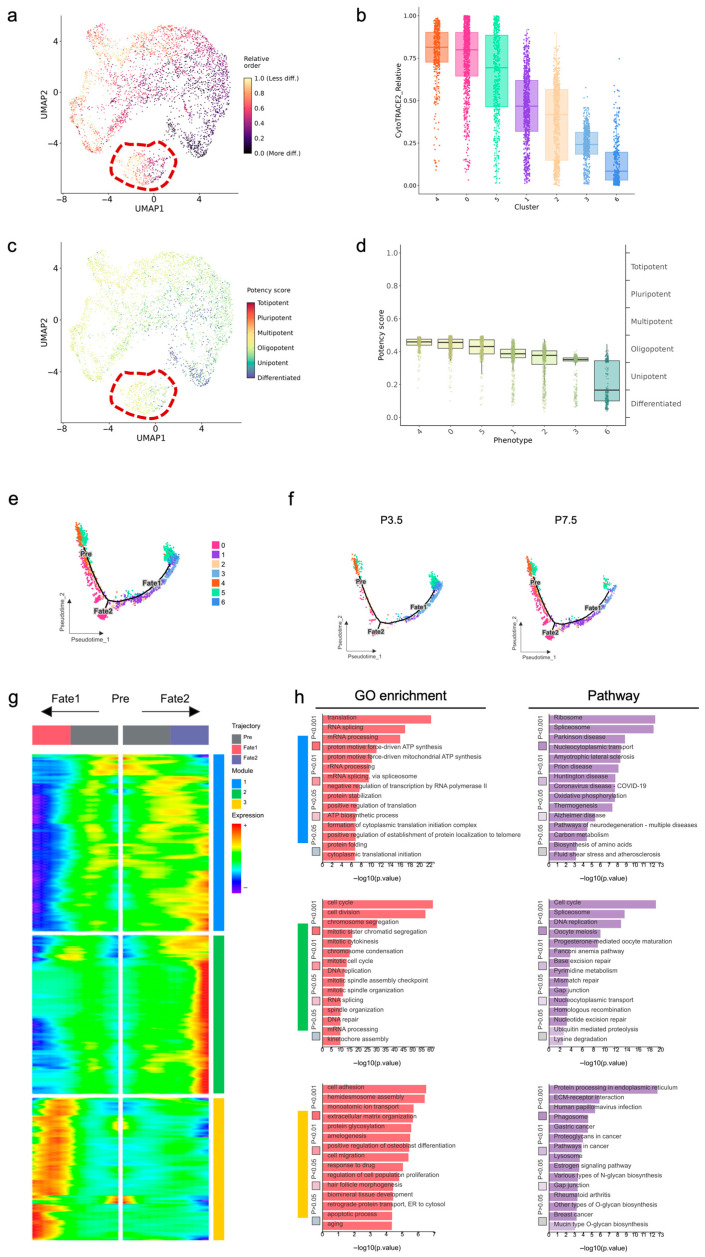
Developmental potency and lineage trajectory of dental epithelial subsets of mouse molars at P3.5 and P7.5: (**a**) UMAP plot visualizing computationally predicted cellular stemness with a value of 1.0 representing the least differentiated state; (**b**) box plot of cellular stemness across clusters; (**c**) UMAP plot visualizing subsets’ potency referring to categorized six distinct types with potency score (high: red; low: blue); (**d**) box plot of developmental potency across clusters; (**e**) scatter plot visualizing cell distribution across pseudotime; (**f**) scatter plot visualizing cell distribution across pseudotime at P3.5 and P7.5 separately; (**g**) heatmap displays expression patterns of key genes associated with fate decision points at branching nodes; (**h**) GO enrichment and pathway analysis of designated gene modules.

Having established the transcriptional heterogeneity and functional identities of the epithelial subpopulations, we next sought to construct their developmental lineage relationships and dynamics. To accurately reconstruct the epithelial developmental trajectory, we first determined the intrinsic differentiation potency of each subpopulation using CytoTRACE2. The predicted hierarchy of cellular differentiation states (Figure 7a,b) positioned Cluster 4 as the most potent progenitor, with a progressive loss of stemness through Clusters 0, 5, 1, 2, and 3, culminating in Cluster 6 as the most differentiated compartment. Based on the computational potency assessment, the epithelial subpopulations exhibit a clear hierarchical organization along the differentiation continuum (Figure 7c,d). Clusters 4, 0, and 5 resided within the oligopotent progenitor zone, while Clusters 1 and 2, despite showing declining potency scores, maintained oligopotent characteristics. Cluster 3 localized near the unipotent boundary, indicating progressive lineage restriction, whereas Cluster 6 occupied the differentiated cell compartment. Interestingly, Cluster 5 exhibited intrinsic heterogeneity, containing intermixed subpopulations of both high and low stemness (Figure 7a,c). Guided by the defined potency hierarchy of epithelial subpopulations, we proceeded to reconstruct their temporal sequence of fate commitment using pseudotime analysis. A bi-potent trajectory originated from a common progenitor pool (Clusters 4, 0, and 5) and segregated into two distinct fate branches (Figure 7e). The Fate 1 branch terminated in the proliferative Cluster 0, representing a self-renewal pathway essential for the maintenance and growth of the epithelial cells. The Fate 2 branch progressed through transitional states (Clusters 1, 2, and 3) and ended up with terminal differentiation, primarily in Cluster 6. Notably, Cluster 5 spanned a broad section of the Fate 2 branch, containing cells that ranged from early progenitor states to nearly fully differentiated cells, underscoring its high internal heterogeneity. The trajectories of P3.5 and P7.5 separated (Figure 7f), revealing a temporal shift in cell fate distribution. At P7.5, Cluster 0 in Fate 1 and Clusters 1 and 2 in Fate 2 expanded significantly, indicating robust progenitor proliferation and transitional state amplification. In contrast, Clusters 3 and 6 in Fate 2 were markedly diminished, reflecting attrition of enamel formation related differentiated populations. The branching event is marked by the divergence of expression patterns (Figure 7g), highlighting genes that drive lineage commitment. Functional annotation and pathway analysis of the designated gene modules revealed significant enrichments in multiple biological categories (Figure 7h). Module 1 showed significant enrichment in translation, RNA splicing, and spliceosome pathways, indicating its role in post-transcriptional regulation and protein synthesis. Module 2 was strongly associated with cell cycle, cell division, and DNA replication, highlighting its involvement in proliferative activities. Module 3 demonstrated enrichments in cell adhesion, extracellular matrix organization, and ECM-receptor interactions, suggesting its function in structural integrity and cell–matrix communication. These results delineate a clear functional partition, with Module 1 regulating basic cellular processes, Module 2 driving proliferation, and Module 3 maintaining tissue architecture, to collectively support epithelial development and lineage commitment during root formation.

**Figure 8:**
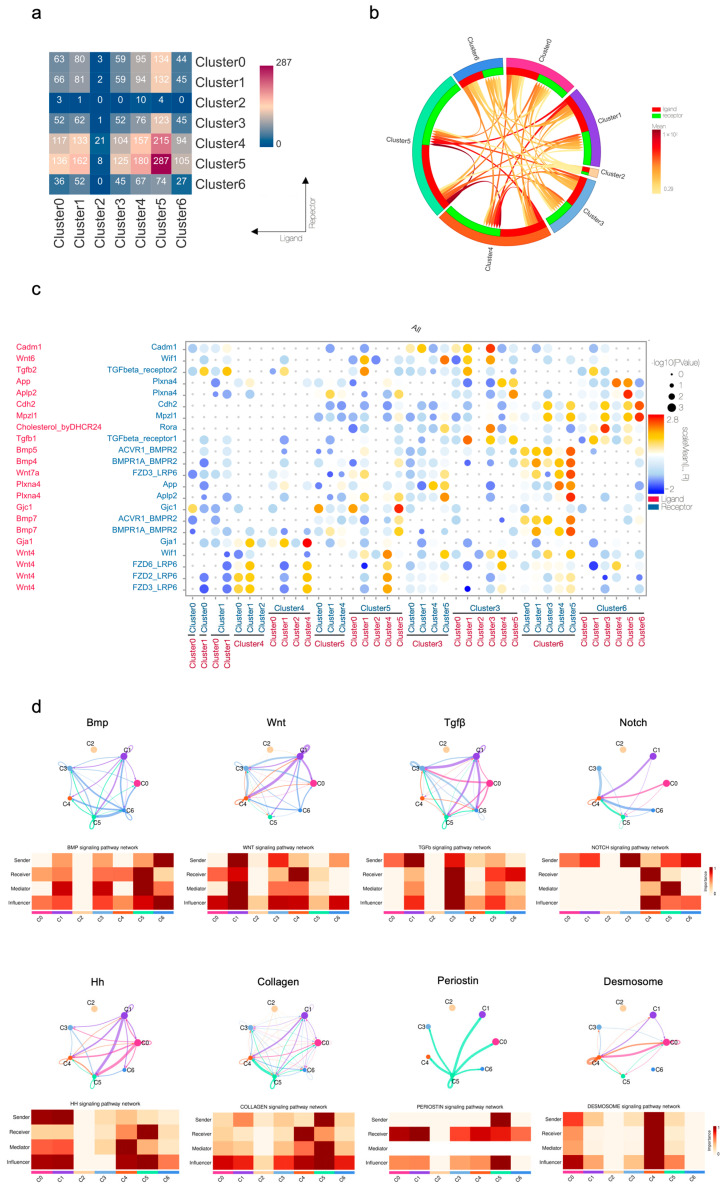
Intercellular communication among the epithelial subclusters of mouse molar at P3.5 and P7.5: (**a**) heatmap quantifying the number of significant ligand–receptor pairs between each cluster; (**b**) circos plot with chordal graph visualizing the overall strength of intercellular communication; (**c**) bubble plot depicting the strength, significance, and specificity of ligand–receptor interactions (*p* value ≤ 0.01, Rank ≥ 0.375, Mean ≥ 0.233); (**d**) circos plot and heatmap revealing the specific role of epithelial cells in signaling pathway network analysis.

With epithelial subcluster identities and lineage relationships defined, we then probed the specific ligand–receptor interactions that mediate their cross-communication. Globally, Clusters 4 and 5 demonstrated the most extensive signaling activity, exhibiting both the highest quantity (Figure 8a) and intensity (Figure 8b) of ligand–receptor interactions among all epithelial subclusters. Specifically, detailed information (Figure 8c) conveyed that intercellular interactions among epithelial subclusters reveal a predominant enrichment in core morphogenetic signaling pathways, particularly Bmp, Wnt, and Tgf-β, along with substantial involvement of cell junction-associated communications. Analysis of key signaling pathways revealed specific communications among epithelial subpopulations (Figure 8d). The foundational morphogenetic gradients are established by Bmp (from Cluster 0 to 5), Wnt (from Cluster 1 to 4), and Tgf-β (from Cluster 1 to 3) pathways, working in concert with Hedgehog signaling (from Cluster 0/1 to 5) to maintain progenitor niches and Notch signaling (from Cluster 3 to 4) to refine cell-fate decisions. Beyond soluble factors, we identified crucial structural communication through collagen (from Cluster 5 to 4) and periostin (from Cluster 5 to 1) signaling, indicating Cluster 5’s dual role as both a signaling hub and extracellular matrix organizer. Notably, autocrine desmosome signaling in Cluster 4 provides mechanical stability while potentially sustaining its progenitor characteristics. This integrated network demonstrated how epithelial subpopulations assume specialized roles, that is, Clusters 0/1 functioning as primary signaling centers, Cluster 5 as a matrix-organizing hub, and Cluster 4 as both a signaling integrator and structural maintainer, to collectively drive the complex process of root formation through precisely coordinated cell–cell communications.

**Figure 9:**
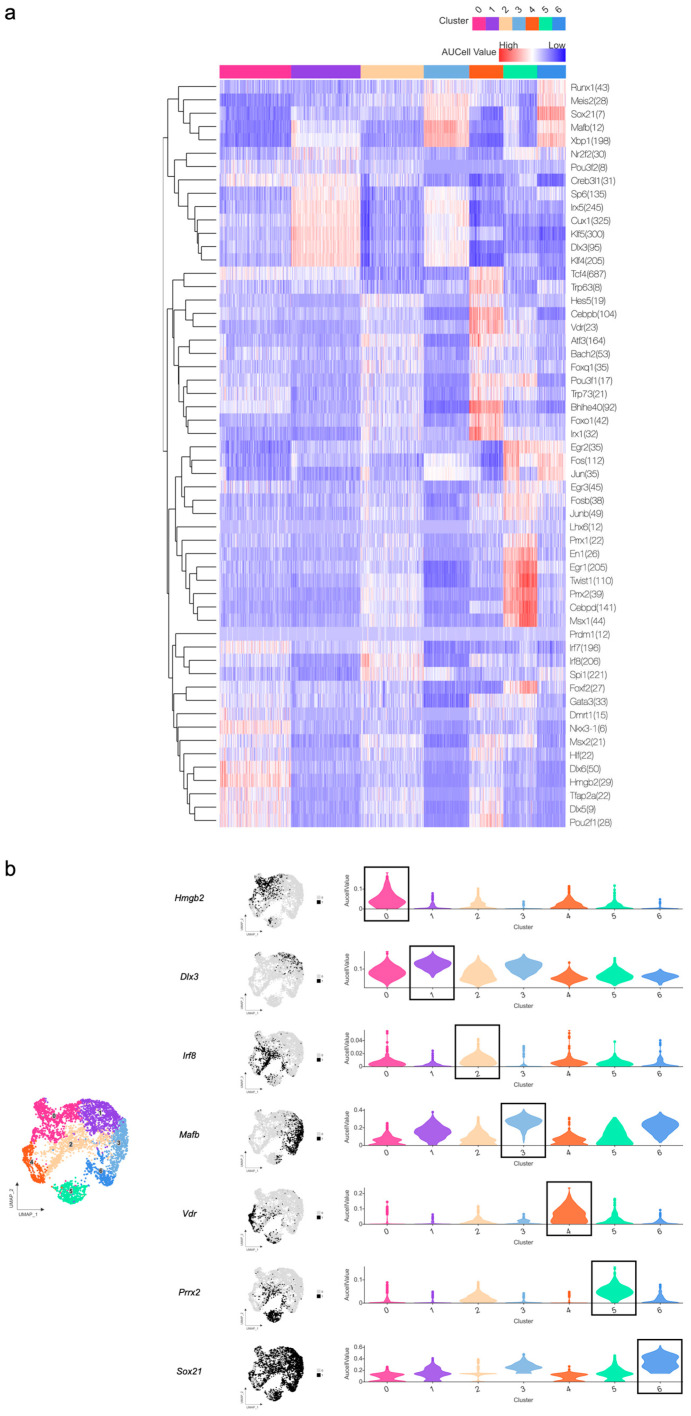
Transcription factor regulatory activity across epithelial subsets of mouse molar at P3.5 and P7.5: (**a**) heatmap displaying the average regulatory activity of TFs across distinct epithelial subclusters; (**b**) distribution and expression of the most dominant TFs in each epithelial subcluster.

With the epithelial signaling network mapped, we turned to identify the mechanistic drivers of fate determination through transcription factor (TF) analysis using SCENIC. The heatmap displayed the average regulatory activity of TFs across distinct cell clusters (Figure 9a). Binary UMAP, along with a violin plot, revealed that the distribution and expression of the most dominant TFs of each subcluster were remarkably concordant with unsupervised clustering boundaries, which implied their specifically restricted activity patterns in the regulation of fate determination (Figure 9b).

**Figure 10:**
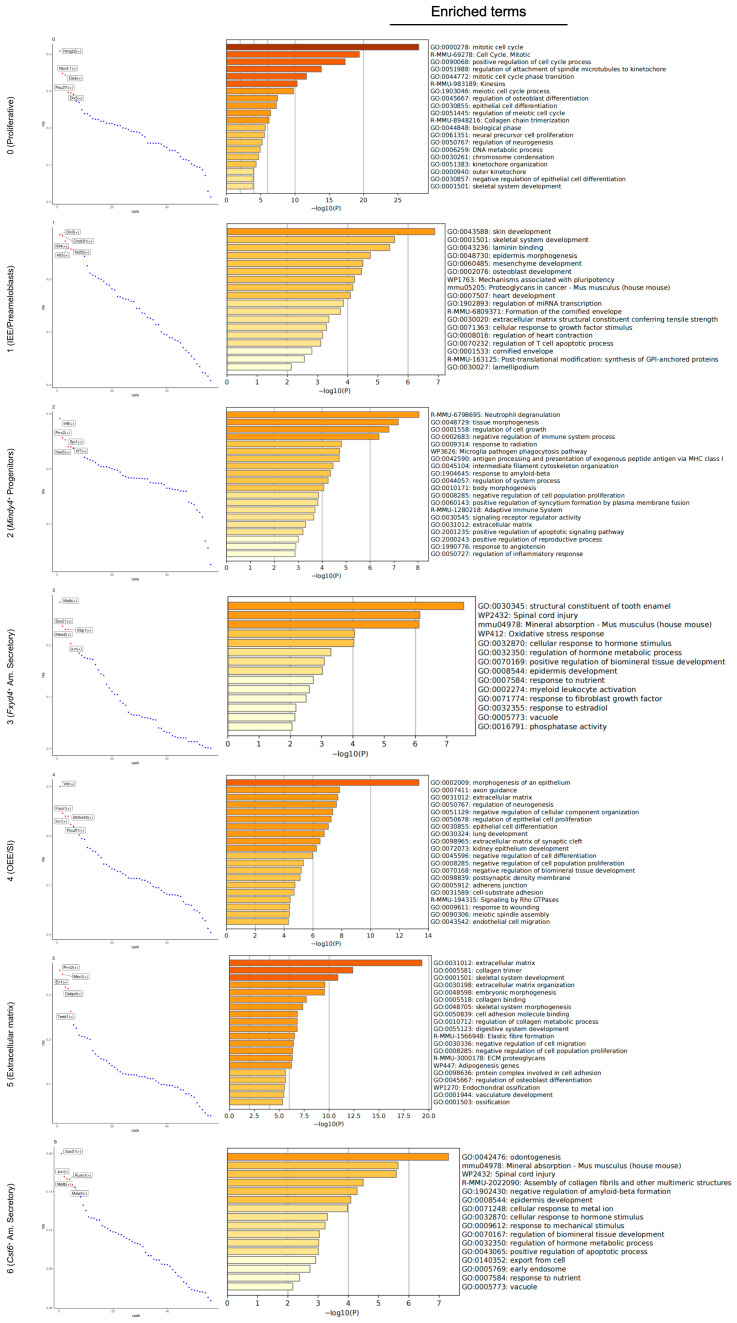
Identification of dominant TFs of epithelial subsets and functional analysis of their predicted target genes in mouse molar at P3.5 and P7.5. Top 5 key TFs of each epithelial subcluster and functional analysis of correspondingly predicted target genes (top 20 of each TF ranked by importance) in each epithelial subcluster.

Subsequently, we identified the top five TFs for each epithelial subpopulation and performed functional enrichment analysis on their predicted target genes (Figure 10). Cluster 0 appeared to be highly enriched for genes that control the cell cycle (both mitotic and meiotic), with a strong emphasis on the precise mechanisms of chromosome segregation. The biological themes for Cluster 1 were strongly associated with development, morphogenesis, and the regulation of these processes through extracellular matrix (ECM) interactions and signaling pathways. Cluster 2 was characterized by a complex gene set that orchestrates defense and response mechanisms by immune surveillance involving both the immediate response (neutrophil degranulation, phagocytosis) and the longer-term adaptive immunity (antigen presentation). Cluster 3 represented a gene program specialized in mineralized tissue formation and maintenance (teeth, bones). The enrichment terms of Cluster 4 strongly emphasized epithelial morphogenesis, dedicated to orchestrating structure and organization. The terms of Cluster 5 were overwhelmingly and specifically associated with the formation, organization, and function of the ECM, with a primary focus on skeletal development and connective tissue morphogenesis. Cluster 6 centered on tooth development and biomineralization. Based on the enrichment analysis, the target genes predicted to be regulated by the key TFs of the seven epithelial clusters revealed a clear biological narrative. Cell cycle and division genes (Cluster 0) were functionally distinct from programs driving tissue morphogenesis (Clusters 1, 2, and 4), which were in turn separate from specialized gene sets governing extracellular matrix assembly and mineralization (Clusters 3, 5, and 6).

### 2.3. Characterization of a Distinct ECM-Associated Epithelial Subpopulation

Building upon our comprehensive analysis of the mouse molar epithelium during the crown–root transition, we identified Cluster 5 as a unique subpopulation which had exhibited four key characteristics: (1) elevated expression of core ECM components and regulatory factors; (2) significant enrichment in ECM organization and mineralization pathways; (3) strategic positioning as both sender and receiver in epithelial communication networks; (4) high internal heterogeneity, with its cells distributed from early progenitor states to nearly fully differentiated cells.

**Figure 11:**
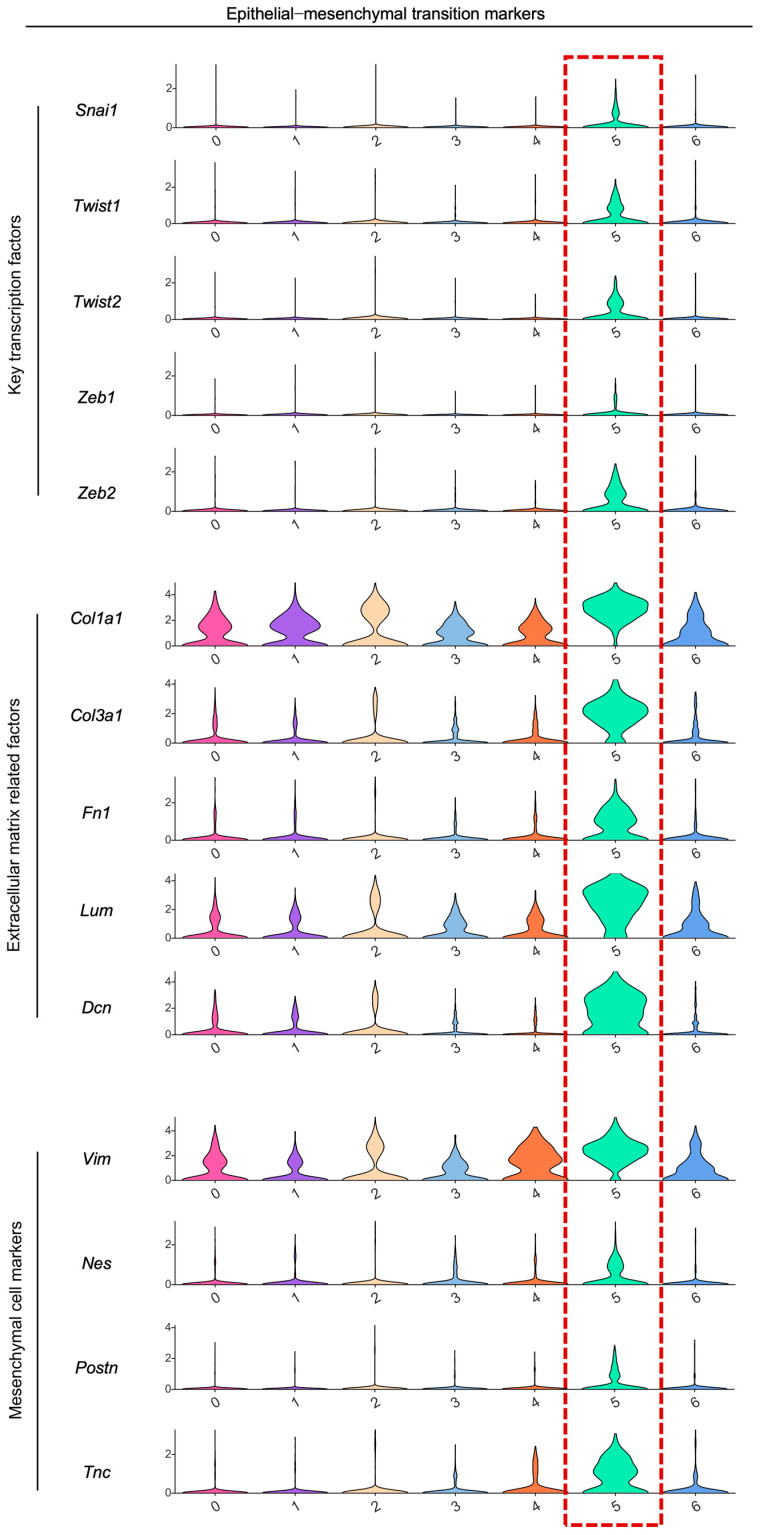
Expression of epithelial–mesenchymal transition markers within the dental epithelium of mouse molars at P3.5 and P7.5.

Even more intriguing was that Cluster 5 exhibited not only features of epithelial cells but also a signature of mesenchymal cells via epithelial–mesenchymal transition (EMT). Cluster 5 demonstrated significantly elevated expression levels of EMT-related key TFs (*Snai1*, *Twist1*, *Twist2*, *Zeb1*, and *Zeb2*), pivotal ECM-associated factors (*Col1a1*, *Col3a1*, *Fn1*, *Lum*, and *Dcn*), and established dental mesenchymal markers (*Vim*, *Nes*, *Postn*, and *Tnc*) compared to all the other epithelial subpopulations (Figure 11). Given above, we pursued further investigation into Cluster 5 to elucidate its specific functions in orchestrating the microenvironment during root formation.

**Figure 12:**
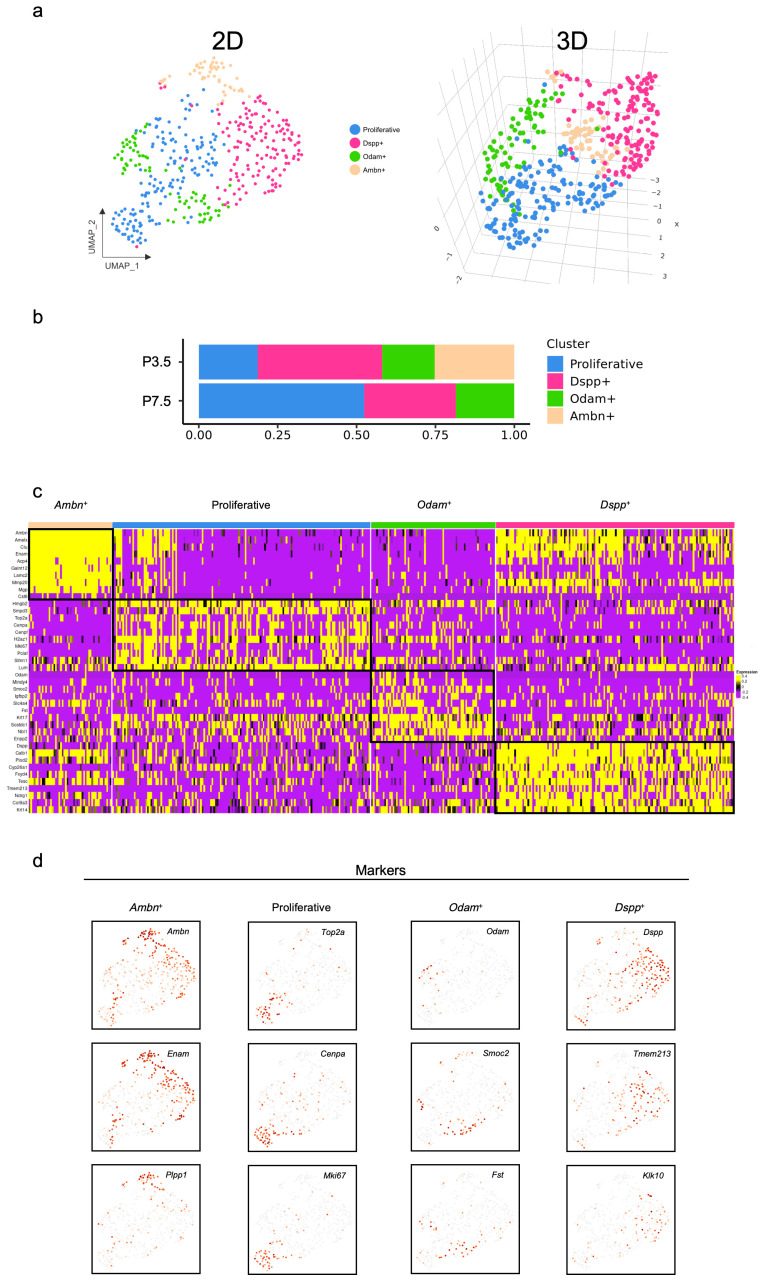
Single-cell transcriptome sequencing and integration analysis of extracellular matrix-associated subpopulation in mouse molars at P3.5 and P7.5: (**a**) UMAP plot visualizing the reclustering from ECM-associated subcluster of dental epithelium in mouse molars, both in 2D and 3D format; (**b**) relative proportions indicating different cell clusters in mouse molars; (**c**) heatmap of gene expression for each cell type of mouse molars; (**d**) expression patterns of the marker genes for the main cell types on the UMAP plot.

For deeper characterization, the 425 cells comprising Cluster 5 were isolated for subsequent detailed analysis. Four distinct subpopulations were identified (Figure 12a). Proportional analysis (Figure 12b) of the ECM-associated epithelial subclusters revealed that by P7.5, the proliferative cluster expanded substantially, the *Dspp*^+^ cluster showed moderate reduction, the *Odam*^+^ cluster remained relatively stable, and the *Ambn*^+^ cluster was completely absent, indicating the termination of crown ameloblast differentiation and the transition to root-specific epithelial structures. The discrete transcriptional identities of the ECM-associated epithelial subclusters were elucidated by a heatmap visualizing the top ten DEGs specific to each (Figure 12c). Specific markers (Figure 12d) of the ECM-associated epithelial subclusters were as follows: proliferative cluster (*Top2a*, *Cenpa*, and *Mki67*), *Dspp*^+^ cluster (*Dspp*, *Tmem213*, and *Klk10*), *Odam*^+^ cluster (*Odam*, *Smoc2*, and *Fst*), and *Ambn*^+^ cluster (*Ambn*, *Enam*, and *Plpp1*).

**Figure 13:**
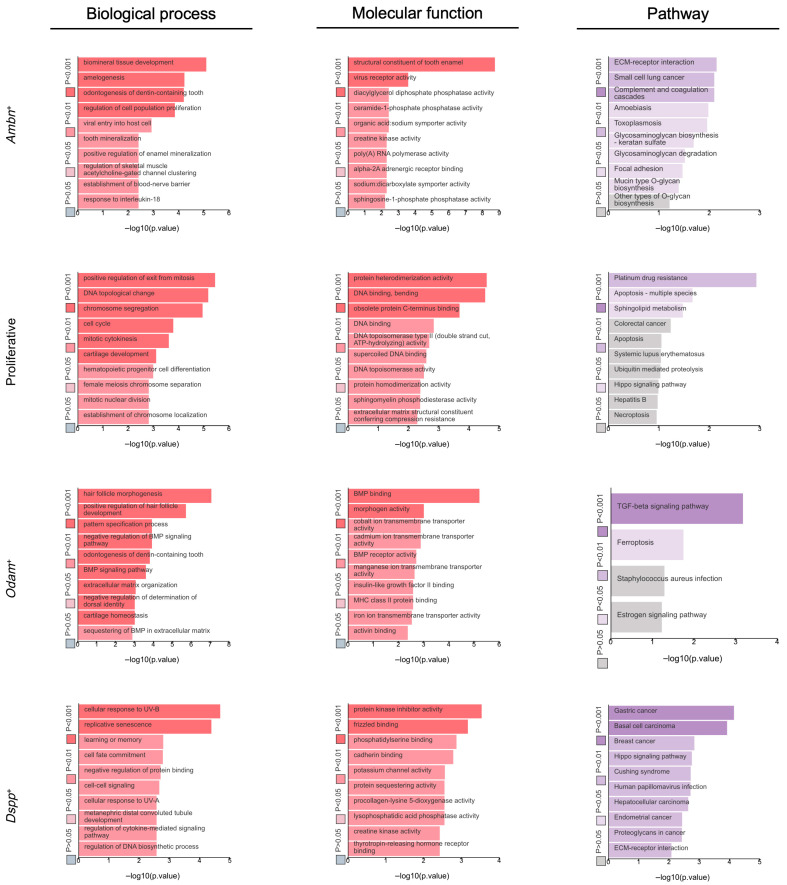
GO and KEGG pathway analyses of ECM-associated dental epithelial subsets in mouse molars at P3.5 and P7.5. log2FC ≥ 1, adj *p* value ≤ 0.01.

The enrichment analysis of the DEGs described specific biological processes (Figure 13). Terms in the *Ambn*^+^ cluster highlighted a central theme of dental and craniofacial development, specifically involving amelogenesis and odontogenesis of dentin-containing teeth. This specialized tissue formation was underpinned by broader mechanisms of biomineralization, including bone mineralization and the positive regulation of extracellular matrix organization. The proliferative cluster was fundamentally defined by cell division, encompassing core cell cycle events, such as mitotic nuclear division, chromosome segregation, mitotic cytokinesis, and the positive regulation of exit from mitosis. The *Odam*^+^ cluster was characterized by its role in developmental morphogenesis and patterning, particularly in hair follicle morphogenesis and odontogenesis of dentin-containing teeth. These processes were guided by key signaling pathways, including the Bmp signaling pathway, the Tgf-β signaling pathway, and the negative regulation of the Wnt signaling pathway. The *Dspp*^+^ cluster exhibited a functional profile centered on environmental stress response, particularly to UV-A and UV-B radiation, and cellular adaptation, through mechanisms like senescence. This was coupled with a role in developmental signaling and fate determination, involving cell–cell communication and cell fate commitment.

**Figure 14:**
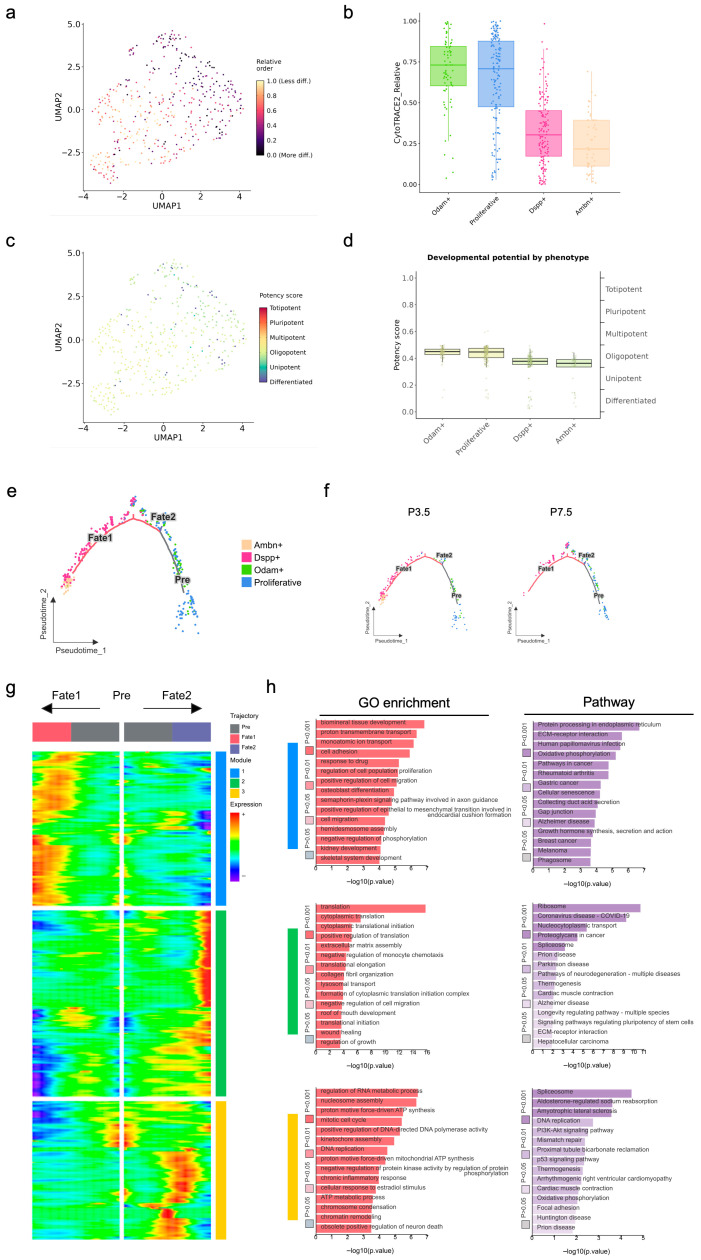
Developmental potency and lineage trajectory of ECM-associated dental epithelial subsets in mouse molars at P3.5 and P7.5: (**a**) UMAP plot visualizing computationally predicted cellular stemness with a value of 1.0 representing the least differentiated state; (**b**) box plot of cellular stemness across clusters; (**c**) UMAP plot visualizing subsets’ potency referring to six distinct categorized types with potency score (high: red; low: blue); (**d**) box plot of developmental potency across clusters; (**e**) scatter plot visualizing cell distribution across pseudotime; (**f**) scatter plot visualizing cell distribution across pseudotime at P3.5 and P7.5 separately; (**g**) heatmap displays expression patterns of key genes associated with fate decision points at branching nodes; (**h**) GO enrichment and pathway analysis of designated gene modules.

To better understand the intrinsic heterogeneity of the ECM-associated epithelial cells, we performed an integrated analysis of their differentiation status, stemness assessment, and developmental trajectory. The *Odam*^+^ cluster and proliferative cluster were less differentiated (Figure 14a,b), while the *Dspp*^+^ cluster and *Ambn*^+^ cluster were closely abutting on the boundary to a unipotent state (Figure 14c,d). Similarly to the developmental pattern of dental epithelium, the trajectory of ECM-associated subsets displayed two fate branches originating from a common progenitor state comprising proliferative cells and *Odam*^+^ epithelial cells (Figure 14e). The Fate 1 branch maintained the proliferative cluster and *Odam^+^* population for self-renewal, supporting epithelial continuity and structural integrity. The Fate 2 branch progressed through *Dspp*^+^ intermediate states and terminated at the *Ambn*^+^ cluster for biomineralization, driving enamel matrix formation and hard tissue development. The *Ambn*^+^ cluster within the Fate 1 branch was undetectable by P7.5 (Figure 14f), reflecting the developmental termination of enamel formation function and the shift from crown- to root-formation programs. The trajectory branching point, marked by divergent gene expression, identified key lineage commitment drivers (Figure 14g). Functional analysis of these gene modules confirmed their roles in distinct biological processes (Figure 14h). Module 3 established nuclear control through RNA metabolism and cell cycle regulation (regulation of RNA metabolic process, nucleosome assembly, mitotic cell cycle, and DNA replication). Module 2 enabled protein synthesis via translational machinery (translation, cytoplasmic translation, cytoplasmic translational initiation, and positive regulation of translation). Module 1 executed extracellular matrix mineralization through biomineral development and osteoblast differentiation (biomineral tissue development, cell adhesion, positive regulation for cell migration, and osteoblast differentiation). These modules formed a coordinated regulatory cascade, that is, Module 3 maintained transcriptional and epigenetic control, Module 2 provided translational capacity, and Module 1 executed extracellular matrix organization and mineralization, collectively driving epithelial differentiation and hard tissue formation.

## 3. Discussion

### 3.1. Deconstructing Dental Epithelium: A Heterogeneous Orchestrator

Our study deconstructed the dental epithelium from a seemingly uniform tissue into a complex, heterogeneous ecosystem of specialized cell states. We provided a transcriptional roadmap, which revealed a structured lineage hierarchy originating from multipotent progenitors and bifurcating into self-renewing and differentiating trajectories. Our data showed that the epithelium is a hub for key morphogenetic signals like Tgf-β, Bmp, and Wnt, and we identified an intricate internal network of cross-communication among distinct subclusters.

Collectively, our analyses support a model in which distinct subclusters contribute specialized transcriptional programs during the crown-to-root transition. The progenitor compartment, including Cluster 4 and the proliferative Cluster 0, shows stem/progenitor-associated features consistent with epithelial progenitor behavior described in the cervical loop [30,31,32]. Cluster 4 is significantly enriched for cell–cell adhesion-related terms, indicating a role in maintaining epithelial structural integrity [33]. Intriguingly, Cluster 4 is also enriched for genes associated with the negative regulation of biomineralization, raising the hypothesis that this subpopulation may contribute to establishing or maintaining an unmineralized periodontal ligament space by limiting hard tissue formation at the epithelial–mesenchymal interface, a function often attributed primarily to mesenchymal components [34]. This potential role is analogous to the barrier function of junctional epithelium at the trans-mucosal interface of dental implants [35]. In addition, we identify Cluster 5 as a unique matrix-organizing hub, exhibiting a hybrid phenotype and orchestrating the microenvironment. Cluster 1 is transcriptionally defined as a key signaling center for morphogenesis, and Cluster 2 may interface with the immune environment. The trajectory culminates in the functional ameloblasts of Clusters 3 and 6. Together, these findings highlight the transcriptional specialization and internal organization of the dental epithelium during the crown-to-root transition.

### 3.2. The ECM-Associated Epithelium: An Active Architect of the Microenvironment

The most striking finding supported by our transcriptional data is the identification of Cluster 5, a unique epithelial subpopulation that co-expresses core epithelial markers together with a robust mesenchymal and ECM signature, including key EMT-associated factors (*Twist1*, *Zeb2*), structural collagens (*Col1a1*, *Col3a1*), and established dental mesenchymal markers (*Postn*, *Vim*). Our result, therefore, provides transcriptional evidence that a subset of epithelial cells adopts a hybrid epithelial–mesenchymal state, contributing to the ongoing discussion regarding the EMT in the HERS [36,37].

Based on its transcriptional profile and communication patterns, we propose that this ECM-associated cluster may function as a pivotal signaling and organizational hub. Its extensive inferred communication via Bmp, Wnt, and Tgf-β pathways, combined with its expression of structural ECM proteins, positions it as a candidate for actively architecting the microenvironment. This suggests a function beyond a solely inductive role, potentially including direct contribution to a mineralization-competent matrix [38]. The internal heterogeneity of this cluster, with its own progenitor (*Odam*^+^) and differentiated (*Ambn*^+^, *Dspp*^+^) substates, further implies a complex role in executing a coordinated program of matrix production and remodeling. Notably, a subpopulation of mesenchymal cells with high-level expression of *LUM*, *MSX1*, and *TWIST1* was identified in human dental epithelium during crown formation [25], hinting at a possible conserved functional module.

### 3.3. Transcriptional Drivers of Fate and Function

The functional specialization of each epithelial subpopulation is reflected in its hardwired transcriptional regulatory program. Our SCENIC analysis revealed that each cluster is governed by a unique set of transcription factors whose predicted target genes align with their biological roles. The clear segregation of regulons controlling cell cycle (Cluster 0), tissue morphogenesis (Clusters 1, 2, and 4), and extracellular matrix assembly/mineralization (Clusters 3, 5, and 6) reinforces the concept of the dental epithelium as a composite of dedicated functional modules.

### 3.4. Advances from the Human Counterpart

The epithelial heterogeneity and dynamic lineage progression we delineate in the mouse molar are strongly echoed and refined by recent single-cell and spatial transcriptomic studies of human teeth. Broadly, comparative atlases confirm the conservation of major dental cell lineages across species while also revealing important species-specific adaptations in progenitor gene expression [26]. This foundational work underscores that the complex epithelial subtypes we identified are not artifacts of the murine model but reflect a fundamental blueprint of odontogenesis. Spatiotemporal atlases of human embryonic development have resolved the dental epithelium into multiple progenitor subclusters, charting their differentiation trajectories, such as the divergence of the SI and ameloblast lineages from a shared precursor pool, and highlighting the critical paracrine signaling from the epithelium to the mesenchyme that guides morphogenesis [27,39]. An integrated multi-omics approach further characterized previously unknown human epithelial subpopulations and discovered a cluster with elevated *TWIST1* and *LUM* expression—a signature reminiscent of the EMT-like, ECM-organizing cluster we describe in mice [25].

The relevance of understanding these epithelial states extends into postnatal homeostasis and disease. The comprehensive atlas of human teeth [40] not only maps cellular heterogeneity but also proposes that functional differences between stem cells are driven by divergent microenvironments—a principle that aligns with the niche-specific signals we find guiding epithelial fate. This concept gains clinical significance from the interactomics of human periodontitis, which shows how adult tooth-associated keratinocytes undergo altered differentiation and mediate inflammatory responses [41], highlighting the pathogenic potential of dysregulated epithelial states. Finally, the integrated resources [42] and the technical strategy combining scRNA-seq with CAGE-seq to identify tooth-enriched epithelial markers [28] provide essential tools for future cross-species validation.

Collectively, the human data confirm the core epithelial biology we describe, while offering a crucial translational framework and identifying conserved regulatory nodes that could be targeted for therapeutic innovation. However, they also highlight important gaps: a human single-cell map specifically targeting the root-forming epithelium (the HERS and its derivatives) is still lacking, and the functional equivalence of subpopulations like our EMT-like Cluster 5 remains to be tested in human contexts.

### 3.5. Future Perspectives

While our atlas provides a comprehensive transcriptional roadmap, it simultaneously opens several avenues for future research to bridge discovery with validation.

A primary limitation is the loss of the native spatial context. Our computational inference of subpopulations, particularly Cluster 5, requires rigorous in situ validation. Future work should employ spatial transcriptomics [25,27] and high-resolution multiplexed imaging to precisely map these states within the developing root architecture.

The isolation and functional interrogation of transcriptionally defined dental epithelial subpopulations remain technically challenging. In most studies, dental epithelial cells are enriched using broadly expressed epithelial surface markers, such as EpCAM [43] and integrin α6/β1 (CD49f/CD29) [44,45]. While effective for isolating the epithelial compartment as a whole, these markers label multiple epithelial states and therefore yield heterogeneous mixtures that span several transcriptional subclusters. In this context, genetic reporter mouse lines provide an important strategy to enrich specific epithelial populations for downstream analyses. For example, *Sox2*-GFP [46,47] has been used to label stem cells in the adult incisor cervical loop, enabling efficient FACS enrichment and functional characterization. Other tools, including *Krt14*-Cre (lineage tracing) [9,48], *Pitx2*-P2A-copGFP (for epithelial cell sorting) [49,50], and *Axin2* reporters (Wnt/β-catenin signaling activity) [51], are powerful for in vivo fate mapping, yet they typically do not achieve isolation of homogeneous populations at subcluster resolution. Together, these observations highlight a recurring bottleneck: transcriptional identity, whether during development or homeostasis, does not readily translate into practical surface marker-based sorting schemes.

Looking ahead, a key step will be to convert transcriptomically defined clusters into experimentally tractable entities. This may be achieved by generating reporter models driven by subcluster-specific markers identified here and/or by identifying corresponding surface antigens that enable prospective enrichment. Such advances would facilitate the transition from correlative atlasing to causal functional validation, and ultimately support more precise cellular engineering strategies for regenerative applications. Our study generates key hypotheses that require causal validation: the role of Cluster 4 in inhibiting mineralization should be tested by the conditional ablation of its key TFs [52], and the microenvironment-organizing function of Cluster 5 should be examined by disrupting its signature EMT or ECM genes.

Our atlas captures the critical early phase of root initiation. However, the role of the epithelium during later stages of root elongation, apex closure, and the establishment of the periodontal attachment apparatus remains an open and clinically significant question [53]: Does the HERS, or its fragmented epithelial rests, continue to exert signaling functions during these later stages? Lineage tracing of our identified progenitor clusters will be essential to track the ultimate fate of these cells and determine if they contribute to the epithelial cell rests of Malassez (ERM) [54]. Understanding the lifelong function of the ERM, potentially in maintaining periodontal homeostasis, could redefine our view of the epithelial legacy in the mature periodontium.

The mouse model is indispensable for dental research, but confirming the conservation of our identified epithelial subpopulations in other species, especially humans, is essential for translational relevance [40]. Future studies should leverage emerging single-cell datasets from developing human teeth to investigate analogous cell states. Comparative analysis could reveal evolutionarily conserved genetic circuits governing root morphogenesis [55], as well as species-specific differences that might underlie variations in root number and shape. Such insights are foundational for the emerging field of regenerative dentistry, as the goal of engineering a biological root likely depends on reactivating conserved epithelial instructional programs.

Ultimately, the most significant translational implication emerging from our transcriptional blueprint lies in the potential to harness these precisely defined epithelial subpopulations for targeted tissue engineering [56]. The paradigm of “one-size-fits-all” cell therapy has shown limited success in regenerating the complex, multi-tissue structure of the tooth root and its periodontium [57]. Our data suggest a more sophisticated, hypothesis-driven approach: using specific, transcriptionally defined cell populations to achieve precise repair of distinct tissue defects. For instance, based on its hybrid EMT and robust ECM-production signature, we hypothesize that the EMT-like Cluster 5 could be a candidate cell source for regenerating the critical bone–cementum interface or for directing the formation of a mineralized scaffold. Conversely, the transcriptional profile of the progenitor-rich Cluster 4, with its enrichment for adhesion and negative regulation of mineralization, implies a potential utility in engineering a stable, non-mineralized periodontal ligament space, a major hurdle in current regenerative protocols. This concept of a “cellular boutique” mirroring advances in other fields, where specific fibroblast subpopulations are being harnessed for targeted wound healing and scar modulation [58], remains a compelling but untested model in the dental counterpart. By moving from heterogeneous mixtures to the precise deployment of functionally validated subpopulations, future studies may aspire to reconstruct the intricate architecture of the tooth attachment apparatus. Future work should focus on developing methods to expand these cell populations in vitro, perhaps through HERS-derived organoid models [59], and testing their combinatorial efficacy in well-established periodontal and root defect models.

In conclusion, this future framework transforms our static transcriptomic snapshot into a dynamic research program. By integrating spatial mapping, functional genetics, cross-species comparison, extended lineage analysis, and translational practice, we can fully elucidate the dental epithelium’s role during root formation and establish a new foundation for understanding developmental biology and pioneering novel regenerative therapies for the tooth and beyond.

## 4. Materials and Methods

### 4.1. Data Sources

The single-cell sequencing datasets of entire mouse molar tissues at postnatal day 3.5 (P3.5) and 7.5 (P7.5) were retrieved from the NCBI Gene Expression Omnibus (accession numbers: GSM5700360 and GSM5700361).

### 4.2. Computational Environment and Reproducibility

All analyses were orchestrated through the NovelBrain Cloud Analysis Platform (NovelBio Co., Ltd., Shanghai, China), which served as a unified interface to execute a series of standard, open-source bioinformatics packages. The core analytical workflows are fully reproducible using the software versions and parameters detailed below. No custom scripts were employed beyond the standard functions of the cited packages. Detailed command logs and session information are available from the corresponding author or the first author upon request.

### 4.3. Data Preprocessing, Integration, and Clustering

Raw gene expression matrices were processed using the SeuratV4 package (https://satijalab.org/seurat/, accessed on 6 November 2025). Cells were filtered based on standard quality control metrics: genes detected per cell (>200 and <10,000). The data were normalized using the SCTransform function. The P3.5 and P7.5 datasets were integrated using Seurat’s canonical correlation analysis (CCA) and anchor-based integration to correct for batch effects. Principal component analysis (PCA) was performed on highly variable genes. Cell clusters were identified using graph-based clustering of the top 10 principal components. A tiered resolution strategy was employed to match the biological scale of each analysis. Parameters were set as follows: the maximum mitochondrial gene percentage was set at 0.2 (20%), no minimum threshold was applied to housekeeping gene expression, and the maximum allowable proportion of digestion-related genes was 0.2. Genes commonly associated with technical or cellular background noise, including mitochondrial genes, erythrocyte genes, and ribosomal genes, were excluded from downstream analyses. For marker gene identification, differential expression analysis was performed using the following cutoffs: a log2 fold change threshold of 0.25 and a minimum expression fraction of 0.1 across cells within the cluster.

A broad resolution of 0.1 was used for the initial pan-tissue clustering to define major lineages. Upon isolation of the epithelial compartment, a moderate resolution of 0.2 was applied to identify principal epithelial subpopulations. Finally, a high resolution of 0.8 was used for the deep subclustering of the ECM-associated epithelium to resolve its fine-scale heterogeneity. The resulting clusters were visualized using uniform manifold approximation and projection (UMAP).

### 4.4. Differential Expression and Functional Enrichment Analysis

Differentially expressed genes (DEGs) for each cluster were identified using the FindAllMarkers function in Seurat (Wilcoxon rank-sum test). Genes with an adjusted *p*-value < 0.01 (Bonferroni correction) and an absolute log2(fold change) > 1 were considered significant. Functional enrichment analyses for Gene Ontology (GO) biological processes and Kyoto Encyclopedia of Genes and Genomes (KEGG) pathways were performed on the cluster-defining DEGs. The top 10 enriched terms per cluster were visualized. Gene modules derived from pseudotime analysis were subjected to similar enrichment analysis, examining the top 15 terms. The predicted target genes of the top transcription factors (from SCENIC analysis) were analyzed using Metascape (http://metascape.org, accessed on 13 November 2025) [60], with a significance cutoff of *p* < 0.01.

### 4.5. Cell–Cell Communication Analysis

Ligand–receptor interactions were analyzed using two complementary tools: CellPhoneDB (v4.1.0) [61] and CellChat (v2.0). Both methods were applied to the normalized Seurat object. Interactions with a *p*-value < 0.05 were considered significant. For high-confidence visualization in bubble plots, only interactions meeting stricter criteria were displayed: *p* ≤ 0.01, interaction rank ≥ 0.375, and mean expression ≥ 0.233. Results from both methods were integrated to identify robust signaling pathways.

### 4.6. Potency Analysis and Developmental Trajectory

Cellular differentiation potency was assessed using CytoTRACE 2 [62] with default parameters. The resulting potency scores were used to orient the developmental trajectory. Pseudotemporal ordering was performed using Monocle 2 (v2.22.0) (https://cole-trapnell-lab.github.io/monocle-release/, accessed on 6 November 2025). The Seurat object was converted to a CellDataSet object, and dimensionality reduction was performed using the DDRTree algorithm. The trajectory was ordered based on genes with differential expression across the epithelium (*q*-value < 0.01). Branch expression analysis modeling (BEAM) was applied to identify genes significantly associated with fate decisions at branch points (*q*-value < 0.01).

### 4.7. Transcription Factor Regulatory Network Analysis

Gene regulatory networks were inferred using the pySCENIC [63] pipeline (v0.9.5), which implements the SCENIC [64] workflow. Transcription factor (TF)–target gene interactions were identified using GRNBoost2 [65]. The analysis was run with the following key parameters: the top 5000 highly variable genes were used as input for GRNBoost2; regulons were inferred with an area under the curve (AUC) threshold of 0.05; and an enrichment score (NES) threshold of 3 was applied for regulon pruning. The resulting binary regulon activity matrix was binarized and used for downstream analysis and visualization.

### 4.8. Immunofluorescence Validation of scRNA-Seq Clusters

All animal experiments were conducted in accordance with the committee guidelines of Sichuan University for animal experiments, which also met the NIH guidelines for the care and use of laboratory animals. Mice at the age of P7.5 were used for the IF test, for the purpose of verifying sequencing analysis results. All procedures were performed in accordance with institutional guidelines and were approved by the Research Ethics Committee of West China Hospital of Stomatology, Sichuan University, under protocol number [WCHSIRB-AT-2025-410].

For tissue preparation, mandibles were dissected from P7.5 C57BL/6 mice, fixed in 4% paraformaldehyde (PFA) at room temperature for 24 h, and decalcified in 10% EDTA (pH 7.4) for 7 days at 37 °C. Tissues were then dehydrated with 15% sucrose solution for 24 h, and, subsequently, 30% sucrose solution for 48 h, embedded in Optimal Cutting Temperature Compound (OCT), and sectioned sagittally with a freezing microtome at a thickness of 10 µm.

For immunofluorescence staining, sections were treated with frozen section specialized antigen retrieval solution from Solarbio (C1035) for 15 min. After washing with PBS, sections were blocked with 10% normal goat serum for 1 h at room temperature. Sections were incubated overnight at 4 °C with primary antibodies diluted in blocking solution. The following primary antibodies were used: rabbit anti-KRT5 (1:100, Huabio, Hangzhou, China, ET1610-43), rabbit anti-KRT14 (1:100, Huabio, ET1610-42), rabbit anti-KRT17 (1:100, Huabio, ET1602-16), rabbit anti-DSC3 (1:100, Huabio, ET7111-26), mouse anti-NECTIN4 (1:100, Huabio, HA723138), rabbit anti-CALB1 (1:100, Huabio, ET1702-54), rabbit anti-CYP26A1 (1:100, Huabio, ET7108-02), mouse anti-DSPP (1:100, Santa Cruz, Dallas, TX, USA, sc-73632), mouse anti-TUBB3 (1:100, Huabio, M0805-8), rabbit anti-LUM (1:100, Huabio, ET7109-24), rabbit anti-COL3A1 (1:100, Abcam, Cambridge, UK, ab7778), rabbit anti-MCM6 (1:100, Huabio, HA721117), rabbit anti-PCNA (1:100, Huabio, ET1605-38), rabbit anti-STMN1 (1:100, Huabio, ET1609-20), and rabbit anti-KI67 (1:100, Huabio, HA721115). After washing, sections were incubated with the appropriate Multi-rAb^®^ CoraLite^®^ Plus 555 Goat Anti Rabbit/Mouse Recombinant Secondary Antibody (H + L) (1:500, Proteintech, Rosemont, IL, USA) for 1 h at room temperature, followed by counterstaining with DAPI to visualize nuclei.

For fluorescence microscopy and image analysis, stained sections were imaged using a Thunder Imager DM6B. Images were acquired at consistent exposure settings across all samples for each channel. For analysis, multi-channel images were processed and merged using Leica LAS X (Wetzlar, Germany). Marker expression patterns were assessed by visual inspection of signal localization and relative intensity across defined anatomical compartments. A minimum of three biological replicates (molars from different mouse mandibles) were conducted.

## 5. Conclusions

This study provides a definitive high-resolution atlas that fundamentally redefines the role of the dental epithelium during the crown-to-root transition. By identifying the specific transcriptional codes and signaling dialogues of these epithelial modules, we provide a mechanistic explanation for how the epithelium simultaneously interacts with surrounding cells and conducts differentiation within its own scope. The information could provide a foundational framework that links developmental biology to precise, cell-based regenerative dentistry.

## Data Availability

Single-cell RNA sequencing data from entire mouse molar tissues at postnatal days 3.5 (P3.5) and 7.5 (P7.5) are publicly available (NCBI GEO: GSM5700360, GSM5700361). Other information can be available from the corresponding author or the first author upon request.
